# Supplementary material for: The spliceosome-associated protein CWC15 promotes miRNA biogenesis in Arabidopsis
Source: Nat Commun. 2024 Mar 16;15:2399. doi: 10.1038/s41467-024-46676-z (PMC10944506; doi:10.1038/s41467-024-46676-z)
Supplement: Supplementary file 1 — Supplementary Information [file 41467_2024_46676_MOESM1_ESM.pdf]

The spliceosome-associated protein CWC15 promotes miRNA biogenesis in Arabidopsis

Banjun Zhou[1, 2], huihui Yu[1, 2], Yong Xue[1, 2], Mu Li[1, 2], Chi Zhang[1, 2] and Bin Yu[1, 2]*

[1] Center for Plant Science Innovation, University of Nebraska-Lincoln, Lincoln, Nebraska 68588–0666, USA

[2] School of Biological Sciences, University of Nebraska-Lincoln, Lincoln, Nebraska 68588–0118, USA

* To whom correspondence should be addressed.
Bin Yu
Tel: +1 402 472 2125;
Fax: +1 402 472 313;
Email: byu3@unl.edu

a

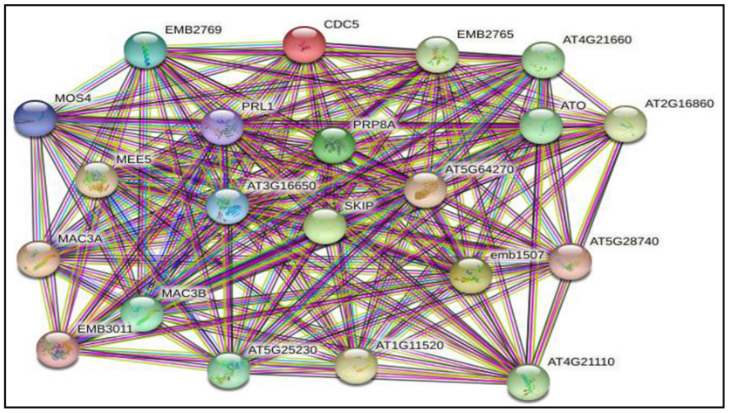

b

MAC components	Other proteins
CDC5	EMB2769 (CWC15)
MAC3A	PRP8A
MAC3B	AT2G16860 (ATSYF2)
MEE5 (MAC11)	AT3G16650 (PRL2)
MOS4	AT4G21110
PRL1	AT5G25230
SKIP (MAC6)	ATO
emb1507 (MAC14)	EMB3011
EMB2765 (MAC7)	AT1G11520
AT5G64270 (MAC18)	
AT5G28740 (MAC9)	

Supplementary Figure 1. Functional gene network of CDC5. Related to Figure 1. **a** Diagram of functional network of CDC5 The network was constructed using STRING program with a high confidence score of 0.7. Nodes and lines indicate genes and functional links, respectively. Line color indicates the type of interaction evidence. Light green: text mining; magenta: experimental data; light blue: association in curated databases; black: co-expression. **b** List of genes involved in RNA metabolism in the CDC5 functional gene network. The genes were listed based on their confidence score from high to low. Source data are provided as a Source Data file.

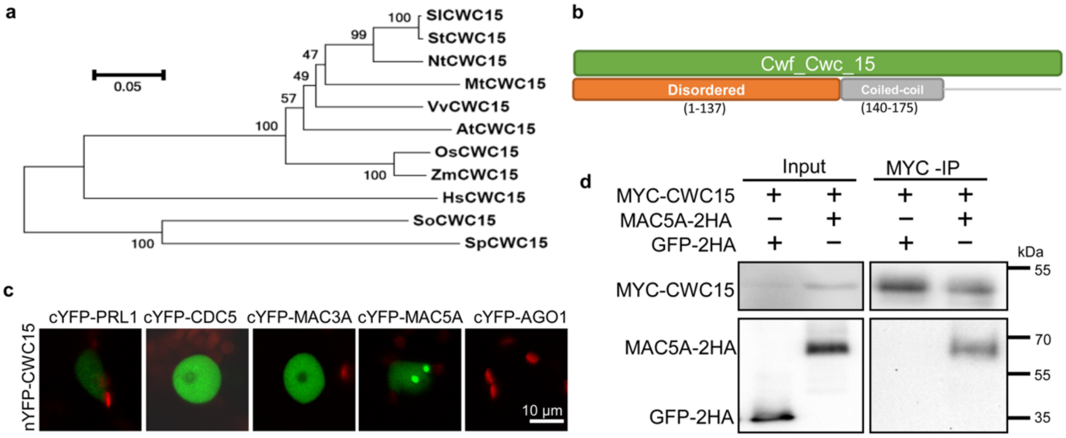

Supplementary Figure 2. CWC15 is a conserved MAC-associated protein. Related to Figure 1. a An unrooted phylogenetic tree of the Cwf_Cwc_15 domain-containing proteins from human, yeast and different plants (Accession numbers: HsCWC15, NP_001350300.1; SoCWC15, XP_013017451.1; SpCWC15, CAA21276.1; SlCWC15, XP_004248344.1; NtCWC15, XP_016459936.1; StCWC15, KAH0721869.1; VvCWC15, CAN74281.1; MtCWC15, XP_003608715.1; OsCWC15, XP_015644216.1; ZmCWC15, NP_001149090.1). **b** Domain architecture of CWC15 protein. **c** *BiFC analysis* of CWC15 with MAC proteins PRL1, CDC5, MAC3A and MAC5A. Paired cYFP- and nYFP-fusion proteins were co-expressed in tobacco leaves. Green color indicates the BiFC signal detected by a confocal microscopy at 48 h after infiltration. Scale bar = 10 μm. **d** Co-IP between MYC-CWC15 and MAC5A-2H. MAC5A-2HA and GFP-2HA were co-expressed with MYC- CWC15 in tobacco leaves, respectively. IPs were performed using anti-MYC antibodies. MAC5A-2HA, GFP-2HA and MYC-CWC15 were detected by western blot. Source data are provided as a Source Data file.

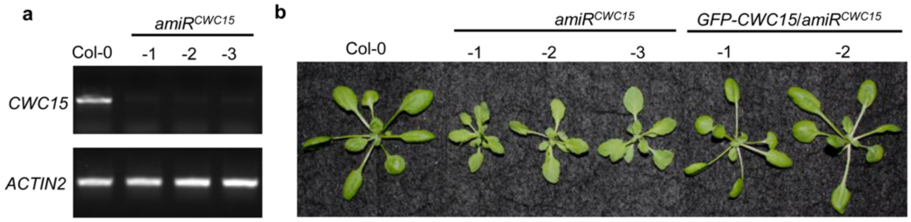

Supplementary Figure 3. Expression analysis and phenotypes of transgenic plants. Related to Figure 1. a RT-PCR analysis of transgenic plants harboring artificial miRNA *amiR*^{CWC15}. *CWC15* transcripts were detected by semi-quantitative RT-PCR**.** *ACTIN2* served as the internal control. **b** Phenotypes of *amiR*^{CWC15} and *amiR*^{CWC15} harboring *pCWC15::GFP-CWC15*. Source data are provided as a Source Data file.

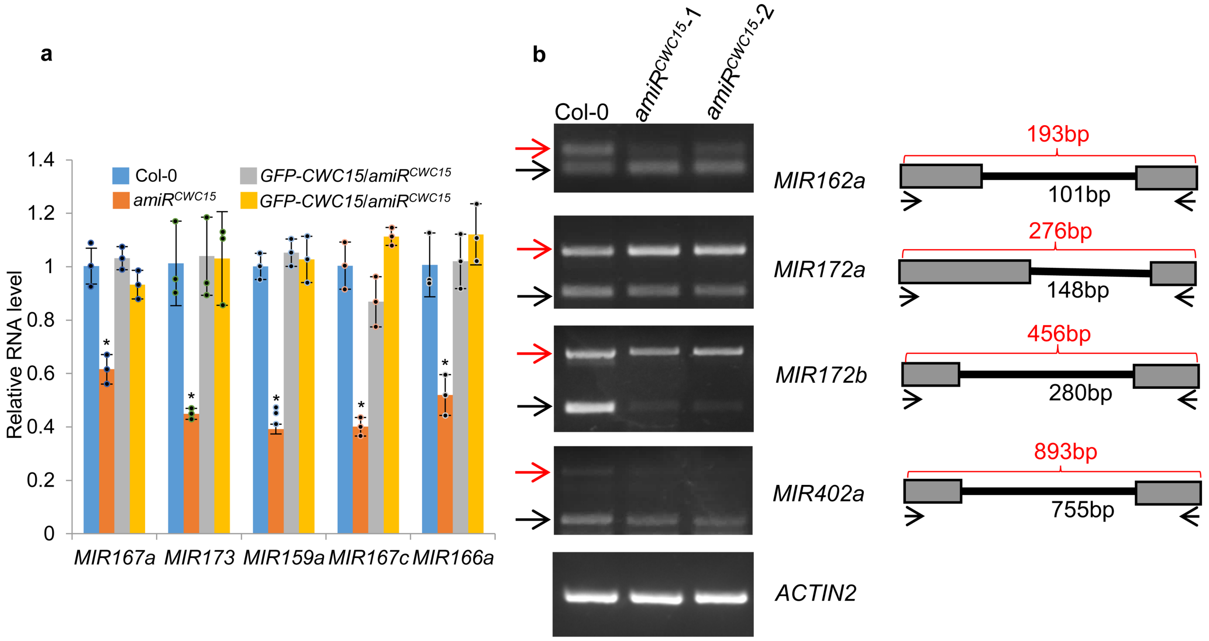

Supplementary Figure 4. Pri-miRNA analyses in various genotypes. Related to Figure 2. a The levels of pri-miRNAs in *PCWC15::GFP-CWC15/amiR^{CWC15}* transgenic plants were detected by RT-qPCR. The levels of pri-miRNAs were normalized to *ACTIN2*. Error bars indicate SD from three biological replicates (n=3) and data are presented as mean values +/- SD. Asterisks indicate significantly reduced expression based on P value (two tailed unpaired t-test; $P < 0.01$): MIR167a, 0.001531; MIR173, 0.003627; MIR159a, 0.000575; MIR167c, 0.000386; MIR166a, 0.003994. **b** Intron-retention of pri-miRNAs analyzed by RT-PCR. Upper arrows indicate the unspliced transcripts. Lower arrows indicate the intron-spliced transcripts. *ACTIN2* served as the loading control. Three replicates were performed and one representative figure was shown for each PCR amplification. Diagram shows the partial regions of several intron-containing pri-miRNAs. Arrows indicate primers used for PCR. The numbers in red indicate the length of PCR products without intron splicing. The numbers in black show the intron length. Source data are provided as a Source Data file.

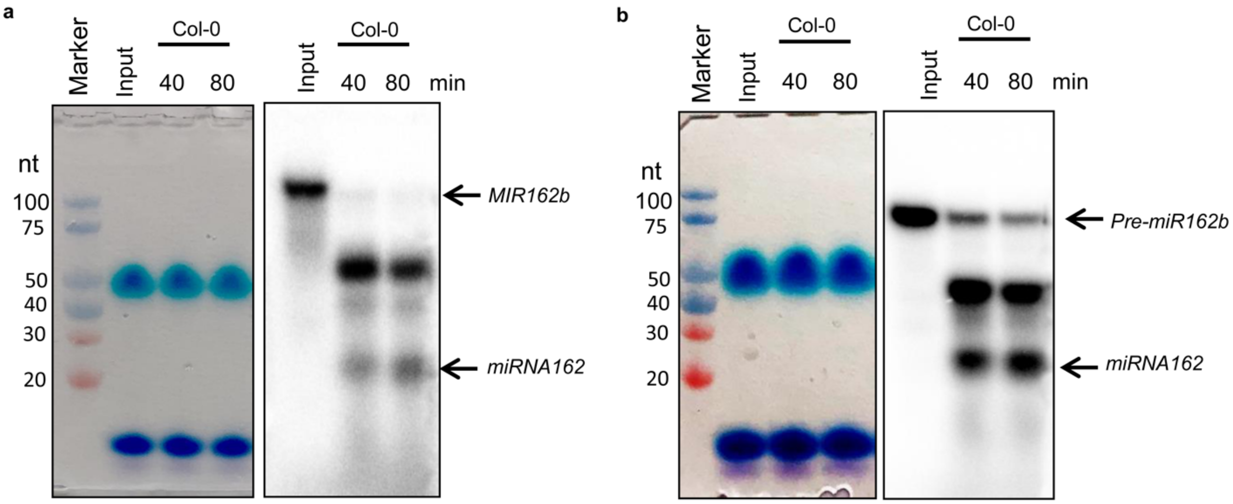

Supplementary Figure 5. Related to Figure 4. **Processing of *MIR162b* and *pre-miR162b*** in proteins extracts from inflorescences of Col. The reactions were stopped at various time points as indicated in the picture. The size difference between MIR162b and pre-pri-miR162b processing intermediates reflects the fact that miR162b is processed from loop-to-base. Source data are provided as a Source Data file.

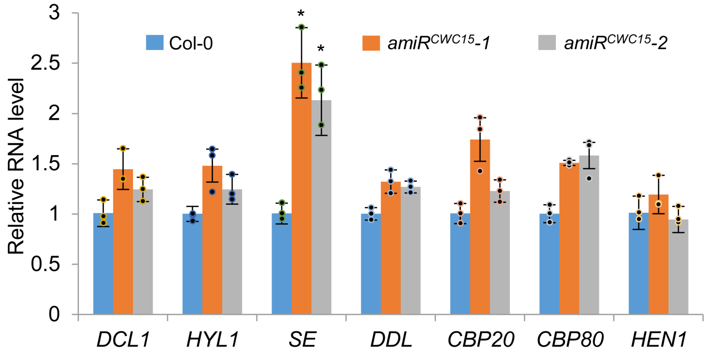

Supplementary Figure 6. Related to Figure 5. The effects of *amiR*^{CWC15} on the transcript levels of key genes involved in miRNA biogenesis determined by RT-qPCR. *ACTIN2* was used as a control. Error bars indicate standard deviation (SD) from three biological replicates (n=3) and data are presented as mean values +/- SD. Asterisks indicate significantly increased SE expression based on P value (two tailed unpaired t-test; $P < 0.01$): 0.002067/ 0.005966. Source data are provided as a Source Data file.

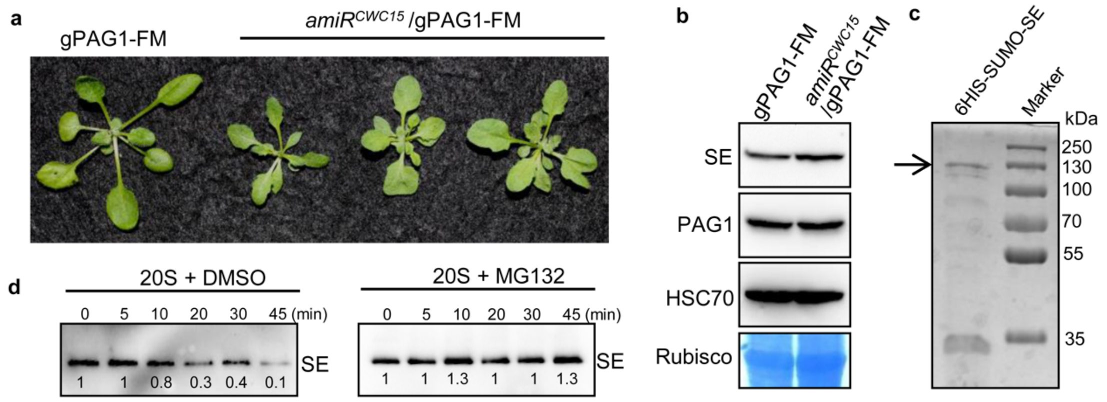

Supplementary Figure 7. Related to Figure 5. ***In vitro*** **reconstitution assays of** 6xHis–SUMO–SE **protein degradation via 20S proteasome. a** The morphological phenotypes of Col and *amiR*^{CWC15} containing *pPAG1::PAG1-FM*. **b** SE and PAG1-FM were detected in Col and *amiR*^{CWC15} containing *pPAG1::PAG1-FM* by western blot. Rubisco stained with Coomassie brilliant blue and HSC70 was used as loading control. **c** Purified 6xHis –SUMO–SE proteins resolved in SDS–PAGE. **d** The degradation of 6xHis–SUMO–SE via 20S proteasome prepared from Col and *amiR*^{CWC15} containing *pPAG1::PAG1-FM* plants. The reaction mixture was applied with either dimethylsulfoxide (DMSO) or 50 µM MG132 and stopped at the indicated time intervals. The numbers below the gels indicate the relative mean signals of SE proteins at different time points that were normalized to those of the proteins at time 0, where the value was arbitrarily assigned a value of 1. Source data are provided as a Source Data file.

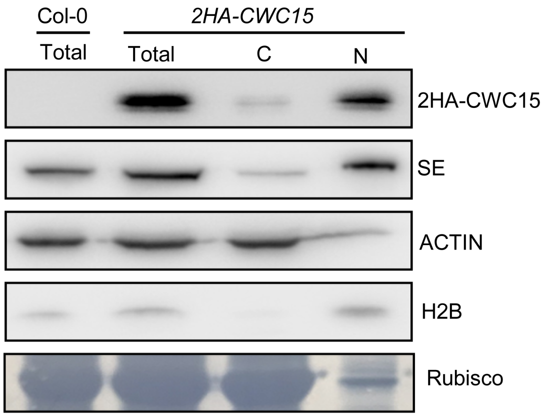

Supplementary Figure 8. Detection of HA-CWC15 in the nuclear and cytoplasmic protein fractions. N: nuclear fraction; C: cytoplasmic fraction. Western blot analysis was conducted with an anti-HA, anti-SE, anti-ACTIN or anti-H2B antibodies. Rubisco stained with Coomassie brilliant blue and H2B detected by anti-H2B antibody were used as controls for the cytoplasmic- and nuclear-specific fractions, respectively. Source data are provided as a Source Data file.

Supplementary Table 1: DNA oligos used in this study.

**Name**	**Sequence (5'-3')**	**Application**
**Primers for constructs**		
a159CWC15-BglII-F	agatcttgatctgacgatggaagATGGCGTTGGTttCCCATAAACA TGAGTTGAGCAGG	pBTEX1300-amiRCWC15
a159CWC15-PSTI-R	ctgcagATGATGGCGTTGGTAACCCATAAAGAAGAGTA AAAGCCATTA	pBTEX1300-amiRCWC15
a159-CWC15-IFPSTI-R	AAAGCAGGGCATGCCTGCAGATGTCGCGGCTCACA CAAC	pBTEX1300-amiRCWC15
PCWC15-PBAeGFM-F	TTGGATCCGGTCTAGAGTAGGATTAACTAGGAAGTC ATGTG	*pCWC15::eGFP-CWC15*
PCWC15-PBAeG-IF-R	TGCTCACCATTCTAGAATCAACCAAGATCGAGATAA GGACG	*pCWC15::eGFP-CWC15*
CWC15-PAB35SGIF-F	GCTGTACAAGCTCGAGATGACGACTGCAGCACGA	*pCWC15::eGFP-CWC15*
CWC15-PAB35SGIF-R	GTTAATTAAGAACCCGGGTCACTTCATGTATCTATG CAGGA	*pCWC15::eGFP-CWC15*
CWC15-PBAFMIF-F	AGGACTTGGGCTCGAGAATGACGACTGCAGCACGA	*p35S::FM-CWC15*
CWC15-PBAFMIF-R	GTTAATTAAGAACCCGGGTCACTTCATGTATCTATG CAGGA	*p35S::FM-CWC15*
N2HA-CWC15-F	tcctctagacccgggATGACGACTGCAGCACGACC	pBTEX1300-2HA-CWC15
N2HA-CWC15-R	AAAGCAGGGCATGCCTGCAGCCCGGGTCACTTCATG TATC	pBTEX1300-2HA-CWC15
HYL1-IFKpnI-F	TTTGGAGAGGACAGGGTACCATGACCTCCACTGATG TTTCC	*p35S::HYL1-2HA*
HYL1-IFStuI-R	GTCGTATGGGTAAGGCCTTGCGTGGCTTGCTTCTGT C	*p35S::HYL1-2HA*
SE-IFKpnI-F	TTTGGAGAGGACAGGGTACCATGGCCGATGTTAATC TTCCT	*p35S:: SE-2HA*
SE-IFStuI-R	GTCGTATGGGTAAGGCCTCAAGCTCCTGTAATCAAT AACGG	*p35S:: SE-2HA*
PAG1-KpnI-F	TTTGGAGAGGACAGGGTACCATGAGTAGCATTGGA ACTGGGTACG	*p35S::PAG1-2HA*
PAG1-StuI-R	GTCGTATGGGTAAGGCCTGTCAGCATCCATCTCCTC G	*p35S::PAG1-2HA*
PAB1-KpnI-F	TTTGGAGAGGACAGGGTACCATGGGAGATAGTCAG TACTCGT	*p35S::PAB1-2HA*
PAB1-StuI-R	GTCGTATGGGTAAGGCCTCTCGACTTCAGCAAGGT	*p35S::PAB1-2HA*
PRP4KA-eGIF-XhoI-F	GCTGTACAAGCTCGAGATGGCGAACGATAAGCAGA TCGA	*p35S::FM-* PRP4KA
PRP4KA-eGFMIF-SmaI-R	GTTAATTAAGAACCCGGGTCACTTGCCAGTGATGAA TGGGT	*p35S::FM-* PRP4KA
CWC15-IFr(MR)173-F	CCGTCGACCTCGAGGGTACCATGACGACTGCAGCAC GAC	pSPYNE(R)173-CWC15<br>pSPYCE (MR)-CWC15
CWC15-IFr(MR)173-R	TCCTACCCGGGAGCGGTACCCTTCATGTATCTATGC AGG	pSPYNE(R)173-CWC15<br>pSPYCE (MR)-CWC15
PRP4KA-BiFC-F	CCGTCGACCTCGAGGGTACCATGGCGAACGATAAG CAGATCGA	pSPYNE(R)173-PRP4KA<br>pSPYCE (MR)-PRP4KA

PRP4KA-BiFC-R	TCCTACCCGGGAGCGGTACCTCACTTGCCAGTGATG AATGGGT	pSPYNE(R)173-PRP4KA pSPYCE (MR)-PRP4KA
Ago1-IFr(MR)173-F	CCGTCGACCTCGAGGGTACCATGGTGAGAAAGAGA AGAACGG	pSPYCE (MR)-AGO1
Ago1-IFr(MR)173-R	TCCTACCCGGGAGCGGTACCGCAGTAGAACATGAC ACGC	pSPYCE (MR)-AGO1
DCl1-IFr(MR)173-F	CCGTCGACCTCGAGGGTACCATGGTAATGGAGGATG AGC	pSPYCE (MR)-DCL1
DCL1-IFr(MR)173-R	TCCTACCCGGGAGCGGTACCAGAAAAAGTTTTATTT AAAAGCTC	pSPYCE (MR)-DCL1
HYL1-IFr(MR)173-F	CCGTCGACCTCGAGGGTACCATGACCTCCACTGATG TTTCCTCT	pSPYNE(R)173-HYL1 pSPYCE (MR)-HYL1
HYL1-IFr(MR)173-R	TCCTACCCGGGAGCGGTACCTGCGTGGCTTGCTTCT GTC	pSPYNE(R)173-HYL1 pSPYCE (MR)-HYL1
SE-IFr(MR)173-F	CCGTCGACCTCGAGGGTACCATGGCCGATGTTAATC TTCCTCC	pSPYNE(R)173-SE pSPYCE (MR)-SE
SE-IFr(MR)173-R	TCCTACCCGGGAGCGGTACCCAAGCTCCTGTAATCA ATAACGG	pSPYNE(R)173-SE pSPYCE (MR)-SE
PRL1-IFr(MR)173-F	CCGTCGACCTCGAGGGTACCATGCCGGCTCCGACGA CG	pSPYCE (MR)-PRL1
PRL1-IFr(MR)173-R	TCCTACCCGGGAGCGGTACCGAAGCGCCTAATCTCC TTTGGTG	pSPYCE (MR)-PRL1
CDC5-IFr(MR)173-F	CCGTCGACCTCGAGGGTACCATGAGGATTATGATTA AGGGAGGTG	pSPYCE (MR)-CDC5
CDC5-IFr(MR)173-R	TCCTACCCGGGAGCGGTACCTGCAGAAGCTTCCATG GC	pSPYCE (MR)-CDC5
MAC3A-IFr(MR)173-F	CCGTCGACCTCGAGGGTACCATGAATTGTGCAATTT CCGGC	pSPYCE (MR)-MAC3A
MAC3A-IFr(MR)173-R	TCCTACCCGGGAGCGGTACCTGAATCTTGTGCTGAA TCTTCA	pSPYCE (MR)-MAC3A
MA5A-IFr(MR)173-F	CCGTCGACCTCGAGGGTACCATGGCTCACAGAATAC TGAGA	pSPYCE (MR)-MAC5A
MAC5A-IFr(MR)173-R	TCCTACCCGGGAGCGGTACCCTGAGACGAACCAGTA GCTGT	pSPYCE (MR)-MAC5A
PAG1-BiFC(R)MR-F	CCGTCGACCTCGAGGGTACCATGAGTAGCATTGGAA CTGGGTACG	pSPYCE (MR)-PAG1
PAG1-BiFC(R)MR-R	TCCTACCCGGGAGCGGTACCTTAGTCAGCATCCATC TCCTCG	pSPYCE (MR)-PAG1
PAB1-BiFC(R)MR-F	CCGTCGACCTCGAGGGTACCATGGGAGATAGTCAGT ACTCGT	pSPYCE (MR)-PAB1
PAB1-BiFC(R)MR-R	TCCTACCCGGGAGCGGTACCTTACTCGACTTCAGCA AGGT	pSPYCE (MR)-PAB1
PBA1-BiFC(R)MR-F	CCGTCGACCTCGAGGGTACCATGGATCTCAATCTCG ATGCACC	pSPYCE (MR)-PBA1
PBA1-BiFC(R)MR-R	TCCTACCCGGGAGCGGTACCTCACATGGCCATTGGT TCAGG	pSPYCE (MR)-PBA1
PBE1-BiFC(R)MR-F	CCGTCGACCTCGAGGGTACCATGAAGCTTGATACTA GTGGGTT	pSPYCE (MR)-PBE1
PBE1-BiFC(R)MR-R	TCCTACCCGGGAGCGGTACCTTATTCGGCTGTTGCTT CCTCC	pSPYCE (MR)-PBE1
**Primers for stem-loop qPCR**		
miR167a-stemloop-RT	GTTGGCTCTGGTGCAGGGTCCGAGGTATTCGCACCA GAGCCAACTAGATC	Stem-loop qPCR
miR167a-Forward	GGCGTCTGAAGCTGCCAGCAT	Stem-loop qPCR

miR173-stemloop-RT	GTTGGCTCTGGTGCAGGGTCCGAGGTATTCGCACCA GAGCCAACGTGATT	Stem-loop qPCR
miR173-forward	GTTGGCTTCGCTTGCAGAGAG	Stem-loop qPCR
miR159a-stemloop-RT	GTTGGCTCTGGTGCAGGGTCCGAGGTATTCGCACCA GAGCCAACTAGAGC	Stem-loop qPCR
miR159a-forward	CGGCGGTTTGGATTGAAGGGA	Stem-loop qPCR
miR167c-stemloop-RT	GTTGGCTCTGGTGCAGGGTCCGAGGTATTCGCACCA GAGCCAACCAAGAT	Stem-loop qPCR
miR167c-Forward	GTTGGCTAAGCTGCCAGCATG	Stem-loop qPCR
mi166a-stemloop-RT	GTTGGCTCTGGTGCAGGGTCCGAGGTATTCGCACCA GAGCCAACGGGGAA	Stem-loop qPCR
miR166a-Forward	TCGCTTCGGACCAGGCTTCA	Stem-loop qPCR
miR156a-stemloop-RT	GTTGGCTCTGGTGCAGGGTCCGAGGTATTCGCACCA GAGCCAACGTGCTC	Stem-loop qPCR
miR156a-Forward	GCGGCGGTGACAGAAGAGAGT	Stem-loop qPCR
U6-stemloop-RT	GTGCAGGGTCCGAGGTTTTGGACCATTTCTCGAT	Stem-loop qPCR
U6-Forward	GGAACGATACAGAGAAGATTAGCA	Stem-loop qPCR
Universal	GTGCAGGGTCCGAGGT	Stem-loop qPCR
**Primers for RT-qPCR**		
UBQ5-qN	GGTGCTAAGAAGAGGAAGAAT	RT-qPCR
UBQ5-qC	CTCCTTCTTTCTGGTAAACGT	RT-qPCR
ACT2-RT-F (AT3G18780) F	CTTGCACCAAGCAGCATGAA	RT-qPCR
ACT2-RT-R (AT3G18780) R	CCGATCCAGACACTGTACTTCCTT	RT-qPCR
DCL1-qF	CGTTGTTATGCGTTTCGACCTTGC	RT-qPCR
DCL1-qR	AACGCTGCGTGAGATACATTTCCTC	RT-qPCR
HYL1 qF	TTGCCTGGATTCTTCAATCGTAAGG	RT-qPCR
HYL1-qR	TAGGTTCTTGCATAATCCCGTTTCG	RT-qPCR
SE-qF	CCACCGCCTCGTAGGGATTACA	RT-qPCR
SE-qR	CCACCATGGTCATACCCAAATCTTC	RT-qPCR
DDL-qF	ATGAGCCCCCAGAGGCTAGAAAAC	RT-qPCR
DDL-qR	CTGCAAGATGGGTGATCCGTAGGAA	RT-qPCR
CBP20-qF	ACCGGCCTATTCGTGTGGATTTTG	RT-qPCR
CBP20-qR	TGCCTTTGTGCTTCGAGTTCCTTC	RT-qPCR
CBP80-qF	TCTGGCAACTGCAACAGTATCCGTA	RT-qPCR
CBP80-qR	GGCAGCAGATGATAGCAATGTTTCG	RT-qPCR
HEN1-qF	TTAGGATGACACCCCCTGATGCTG	RT-qPCR
HEN1-qR	AAAAGCCGCCTCCATTCGTTCTTC	RT-qPCR
GUS-qF	CGATGTCACTCCGTATGTTATTG	RT-qPCR
GUS-qR	CAGTTCTTTCGGCTTGTTGC	RT-qPCR
MIR156a qF	AAGGGGGTCTTCTATCATCAGGA	RT-qPCR
MIR156a qR	TGATTGGAATATGCCCTAAAGAGTG	RT-qPCR
MIR166a qF	GACTCTGGCTCGCTCTATTCA	RT-qPCR

MIR166a qR	TGGTCCGAAGACGCTAAAAC	RT-qPCR
MIR167a qF	TGTTGTGTTTCATGACGATGG	RT-qPCR
MIR167a qR	AGCTCACAAAATCAGACTGAAGA	RT-qPCR
MIR172b qF	GTAGGCGCAGCACCATTAAG	RT-qPCR
MIR172b qR	TTTGTAGCCGTCGATTGTTG	RT-qPCR
MIR159a qF	TCAGGAGCTTTAACTTGCCCTTT	RT-qPCR
MIR159a qR	CACGCTAAACATTGCTTCGGAAT	RT-qPCR
MIR164a qF	CCCTCATGTGCTTGGAAATG	RT-qPCR
MIR164a qR	GCAAATGAGACGGATTTCGTG	RT-qPCR
MIR173 qF	CTTCTTCTCACAAATAAACCCA	RT-qPCR
MIR173 qR	AAGATCTCTAACATTAAATCAT	RT-qPCR
MIR167c qF	TTAAGCTGCCAGCATGATCT	RT-qPCR
MIR167c qR	TCTTCTCCTTCATGCTACAATCA	RT-qPCR
MIR171c qF	ATGTGGATGGAGTTTGGTGTAA	RT-qPCR
MIR171c qR	GTGATATTGGCACGGCTCA	RT-qPCR
MIR156b qF	GCTAGAAGAGGGAGAGATGGTGATTGAG	RT-qPCR
MIR156b qR	GTGAGCACGCACACGCAAAGTTATAGAC	RT-qPCR
qARF8-1F	CAGGGTCGGTCGGGCGATCA	RT-qPCR
qARF8-1R	CCCCTGCTCCCCCATCTTTT	RT-qPCR
qCMT3-1F	ATGCTGAAGATGGCTAAG	RT-qPCR
qCMT3-1R	CATTCCTCACTTGGTAATTC	RT-qPCR
qAPS3-1F	GCGGCGGATTTGCCGAGAGT	RT-qPCR
qAPS3-1R	CCCACGAAGAGGACTAGCCCAAC	RT-qPCR
qCKB3-1F	ATGTACAAGGAACGTAGTGG	RT-qPCR
qCKB3-1R	CTAGATGTGGTGGTGGAAGT	RT-qPCR
qPHV-1F	GGATTTGATTCCGGCAAGTA	RT-qPCR
qPHV-1R	TCTCGACATCTGCGATTCTG	RT-qPCR
qARLPK1-1F	TTATTAGGAGATGTCTGAAGGAT	RT-qPCR
qARLPK1-1R	TCATTTCCTGATCTCTTCAAGCT	RT-qPCR
**Primers for ChIP qPCR**		
Pol II-C1-F	AGTTCAATGGAGAGATGTCGAAATATG	ChIP-qPCR
Pol II-C1-R	AAGAGGAAAAGAAAGAGATGGAGAGA	ChIP-qPCR
cMIR159a-F	TGGCAGGAACGAATAATAATTG	ChIP-qPCR
cMIR159a-R	GAACAGGTGGGTGCATCTGAA	ChIP-qPCR
cMIR173-F	ACTTAAAGCGGCGGTCTCA	ChIP-qPCR
cMIR173-R	GGGTTTATTTGTGAGAAGAAGATCA	ChIP-qPCR
cMIR172b-F	AGGAGAAAAGCAGTGGGATA	ChIP-qPCR
cMIR172b-R	CCTTGGATTCGTGAGGTT	ChIP-qPCR
cMIR171a-F	TGCTTTGGTAGTAGATGAGGTT	ChIP-qPCR
cMIR171a-R	CGTGTGTGGTCAGGTAAGAT	ChIP-qPCR
cMIR168a-F	AACACATTCACATACATTACGTTGG	ChIP-qPCR

cMIR168a-R	TATTTGGAAAAGATTAGAACAGCG	ChIP-qPCR
**Probe sequence for Northern blot**		
miR156	GTGCTCACTCTCTTCTGTCA	Northern blot
miR169	T+CGG+CAA+GTC+ATC+CTT+GGC+TG	Northern blot
miR166	GG+GGA+ATG+AAG+CCT+GGT+CCG+T	Northern blot
miR167	T+AGA+TCA+TGT+TGG+CAG+TTT+CA	Northern blot
miR172	AT+GCA+GCA+TCA+TCA+AGA+TTC+T	Northern blot
miR319	GGG+AGC+TCC+CTT+CAG+TCC+AA	Northern blot
miR390	G+GCG+CTA+TCC+CTC+CTG+AGC+TT	Northern blot
miR173	G+TGA+TTT+CTC+TCT+CGA+AGC+GAA	Northern blot
U6	TCATCCTTGCGCAGGGGCCA	Northern blot
**Primers for *in vitro* RNA processing assay**		
T7miR162b-p3	TAATACGACTCACTATAGGGAAAGAGTGAAGTCGCTGGAG	Pri-miR162b Probe
miR162b-p4	CATGAAGAGCAAGCAGCGCTGGATGC	Pri-miR162b Probe
T7-premiR162bF	TAATACGACTCACTATAGGAGGCAGCGGTTCATCGATC	Pre-miR162b Probe
premiR162bR	CTGGATGCAGAGGTTTATCGATC	Pre-miR162b Probe
