# Supplementary material for: The spliceosome-associated protein CWC15 promotes miRNA biogenesis in Arabidopsis
Source: Nat Commun. 2024 Mar 16;15:2399. doi: 10.1038/s41467-024-46676-z (PMC10944506; doi:10.1038/s41467-024-46676-z)
Supplement: Supplementary file 3 — Description of Additional Supplementary Information [file 41467_2024_46676_MOESM3_ESM.pdf]

Description of Additional supplementary files

Supplementary Dataset 1: miRNA profiling analysis.
