# Supplementary material for: The spliceosome-associated protein CWC15 promotes miRNA biogenesis in Arabidopsis
Source: Nat Commun. 2024 Mar 16;15:2399. doi: 10.1038/s41467-024-46676-z (PMC10944506; doi:10.1038/s41467-024-46676-z)
Supplement: Supplementary file 5 — Reporting Summary [file 41467_2024_46676_MOESM5_ESM.pdf]

nature portfolio

Corresponding author(s): Bin Yu

Last updated by author(s): 02/02/2024



# Reporting Summary

Nature Portfolio wishes to improve the reproducibility of the work that we publish. This form provides structure for consistency and transparency in reporting. For further information on Nature Portfolio policies, see our Editorial Policies and the Editorial Policy Checklist.

## Statistics

For all statistical analyses, confirm that the following items are present in the figure legend, table legend, main text, or Methods section.

| n/a | Confirmed | |
|---|---|---|
| ☐ | ☒ | The exact sample size (*n*) for each experimental group/condition, given as a discrete number and unit of measurement |
| ☐ | ☒ | A statement on whether measurements were taken from distinct samples or whether the same sample was measured repeatedly |
| ☐ | ☒ | The statistical test(s) used AND whether they are one- or two-sided<br>*Only common tests should be described solely by name; describe more complex techniques in the Methods section.* |
| ☒ | ☐ | A description of all covariates tested |
| ☐ | ☒ | A description of any assumptions or corrections, such as tests of normality and adjustment for multiple comparisons |
| ☐ | ☒ | A full description of the statistical parameters including central tendency (e.g. means) or other basic estimates (e.g. regression coefficient) AND variation (e.g. standard deviation) or associated estimates of uncertainty (e.g. confidence intervals) |
| ☐ | ☒ | For null hypothesis testing, the test statistic (e.g. *F*, *t*, *r*) with confidence intervals, effect sizes, degrees of freedom and *P* value noted<br>*Give P values as exact values whenever suitable.* |
| ☒ | ☐ | For Bayesian analysis, information on the choice of priors and Markov chain Monte Carlo settings |
| ☒ | ☐ | For hierarchical and complex designs, identification of the appropriate level for tests and full reporting of outcomes |
| ☒ | ☐ | Estimates of effect sizes (e.g. Cohen's *d*, Pearson's *r*), indicating how they were calculated |

*Our web collection on statistics for biologists contains articles on many of the points above.*

## Software and code

Policy information about availability of computer code

| | |
|---|---|
| Data collection | No software is used for data collection |
| Data analysis | For miRNA-Seq data analysis, short reads were mapped to Araport11 genome using bowtie (v0.12.7). Only uniquely mapped perfect-match reads were kept. HTSeq (v0.6.1p1) was used for read-counting on protein-coding genes (based on Araport11) and mature miRNA (from miRbase) with the strand-specific mode in HTSeq (v0.6.1p1).The gene/miRNA read-count data were normalized with DESeq2(v1.30.1). Differential analysis of miRNA expression between two groups was conducted by DESeq2 (v1.30.1). |

For manuscripts utilizing custom algorithms or software that are central to the research but not yet described in published literature, software must be made available to editors and reviewers. We strongly encourage code deposition in a community repository (e.g. GitHub). See the Nature Portfolio guidelines for submitting code & software for further information.

## Data

Policy information about availability of data

All manuscripts must include a data availability statement. This statement should provide the following information, where applicable:

- Accession codes, unique identifiers, or web links for publicly available datasets
- A description of any restrictions on data availability
- For clinical datasets or third party data, please ensure that the statement adheres to our policy

All Sequencing data used in this study are available in National Center for Biotechnology Information Gene Expression Omnibus under accession codes:

GSM7122973 (https://www.ncbi.nlm.nih.gov/geo/query/acc.cgi?acc=GSM7122973), GSM7122974 (https://www.ncbi.nlm.nih.gov/geo/query/acc.cgi?acc=GSM7122974), GSM7122975 (https://www.ncbi.nlm.nih.gov/geo/query/acc.cgi?acc=GSM7122975) and GSM7122976 (https://www.ncbi.nlm.nih.gov/geo/query/acc.cgi?acc=GSM7122976). Araport11 genome (https://www.arabidopsis.org/download/index-auto.jsp?dir=%2Fdownload_files%2FGenes%2FAraport11_genome_release) was used for miRNA-Seq data analysis.

## Research involving human participants, their data, or biological material

Policy information about studies with human participants or human data. See also policy information about sex, gender (identity/presentation), and sexual orientation and race, ethnicity and racism.

| | |
|---|---|
| Reporting on sex and gender | NA |
| Reporting on race, ethnicity, or other socially relevant groupings | NA |
| Population characteristics | NA |
| Recruitment | NA |
| Ethics oversight | NA |

Note that full information on the approval of the study protocol must also be provided in the manuscript.

# Field-specific reporting

Please select the one below that is the best fit for your research. If you are not sure, read the appropriate sections before making your selection.

☒ Life sciences ☐ Behavioural & social sciences ☐ Ecological, evolutionary & environmental sciences

For a reference copy of the document with all sections, see nature.com/documents/nr-reporting-summary-flat.pdf

# Life sciences study design

All studies must disclose on these points even when the disclosure is negative.

| | |
|---|---|
| Sample size | In this study, two biological replicates for flowers from at least 10 plants were used to profile miRNA-Seq. For greenhouse or chamber plant experiments, all of the experiments on seedlings and flowers were conducted using at least 10 seedlings or plants in three independent replicates. The similar sample sizes have been successfully used by our lab and other lab in many published papers, such as Li, Shengjun, et al., 2022. |
| Data exclusions | No data is excluded |
| Replication | Each genotype, including Wildtype and mutant, has two replicates for RNA-seq. Repeatability of miRNA-seq showed in Fig. 1 and all other replicated experiments described in the manuscript are successful. |
| Randomization | We randomized the position of the pots and plates for the plants to avoid the potential light coverage and intensity variations in the greenhouse and growth chamber. Samples from Wildtype and mutant were randomly collected form the pots and plates with random positions in greenhouse and growth chamber. |
| Blinding | All of the plants need to be clearly labeled and genotyped to avoid any cross-contamination during the harvesting for experiments, which is critical for testing the difference between different plant lines. Therefore, binding sample collection is impossible for this study. We need to clearly verify any difference between wildtype and mutant group. |

# Reporting for specific materials, systems and methods

We require information from authors about some types of materials, experimental systems and methods used in many studies. Here, indicate whether each material, system or method listed is relevant to your study. If you are not sure if a list item applies to your research, read the appropriate section before selecting a response.

## Materials & experimental systems

| n/a | Involved in the study | |
|---|---|---|
| ☐ | ☒ | Antibodies |
| ☒ | ☐ | Eukaryotic cell lines |
| ☒ | ☐ | Palaeontology and archaeology |
| ☒ | ☐ | Animals and other organisms |
| ☒ | ☐ | Clinical data |
| ☒ | ☐ | Dual use research of concern |
| ☐ | ☒ | Plants |

## Methods

| n/a | Involved in the study | |
|---|---|---|
| ☐ | ☐ | ChIP-seq |
| ☐ | ☐ | Flow cytometry |
| ☐ | ☐ | MRI-based neuroimaging |

# Antibodies

| | |
|---|---|
| Antibodies used | anti-GFP (1:1000 dilutions, rabbit polyclonal, ab190584, Abcam), anti-HYL1 (1:5000 dilutions, rabbit polyclonal, AS06136, Agrisera), anti-SE (1:5000 dilutions, rabbit polyclonal, AS09532A, Agrisera), anti-DCL1(1: 1000 dilutions, rabbit polyclonal, AS194307, Agrisera), anti-MYC (1: 5000 dilution, rabbit monoclonal, M4439, Sigma), anti-FLAG (1: 10000 dilutions, mouse monoclonal, A8592, Sigma), anti-HA (1: 5000 dilutions, mouse monoclonal, H6533, Sigma), anti-RPB2 (1: 5000 dilutions, mouse monoclonal, ab817, Abcam) |
| Validation | These antibodies have been used and the results have been published in many high-profiled journals.<br>anti-GFP (1:1000 dilutions, rabbit polyclonal, ab190584, Abcam) was used and cited by Priyanka Singh et al 2021 Mol Cell.<br>anti-HYL1 (1:5000 dilutions, rabbit polyclonal, AS06136, Agrisera), anti-SE (1:5000 dilutions, rabbit polyclonal, AS09532A, Agrisera) and anti-DCL1(1: 1000 dilutions, rabbit polyclonal, AS194307, Agrisera), were used and cited by Hongbo Li et al 2023 EMBO Reports and Agata Stepien et al 2022 The Plant Cell.<br>anti-MYC (1: 5000 dilution, rabbit monoclonal, M4439, Sigma) was used and cited by Chunyuan Zhao et al 2017 Cell Rep. and MohanLyu et al 2019 Development Cell.<br>anti-FLAG (1: 10000 dilutions, mouse monoclonal, A8592, Sigma) was used and cited by Mickaël Lelek et al.2015 Nature communications and Martin Roth et al. 2010 Development.<br>anti-HA (1: 5000 dilutions, mouse monoclonal, H6533, Sigma) was used and cited by Katharina L Willmann et al. 2014 Nature communications and Joanna F McGouran et al 2013 Chemistry & biology.<br>anti-RPB2 (1: 5000 dilutions, mouse monoclonal, ab817, Abcam) was used and cited by Bunch, H. et al 2023 Nature communications and Heuzé, J. et al 2023 The EMBO Journal. |

# Dual use research of concern

Policy information about dual use research of concern

## Hazards

Could the accidental, deliberate or reckless misuse of agents or technologies generated in the work, or the application of information presented in the manuscript, pose a threat to:

| No | Yes | |
|---|---|---|
| ☒ | ☐ | Public health |
| ☒ | ☐ | National security |
| ☒ | ☐ | Crops and/or livestock |
| ☒ | ☐ | Ecosystems |
| ☒ | ☐ | Any other significant area |

## Experiments of concern

Does the work involve any of these experiments of concern:

| No | Yes | |
|---|---|---|
| ☒ | ☐ | Demonstrate how to render a vaccine ineffective |
| ☒ | ☐ | Confer resistance to therapeutically useful antibiotics or antiviral agents |
| ☒ | ☐ | Enhance the virulence of a pathogen or render a nonpathogen virulent |
| ☒ | ☐ | Increase transmissibility of a pathogen |
| ☒ | ☐ | Alter the host range of a pathogen |
| ☒ | ☐ | Enable evasion of diagnostic/detection modalities |
| ☒ | ☐ | Enable the weaponization of a biological agent or toxin |
| ☒ | ☐ | Any other potentially harmful combination of experiments and agents |

# Plants

| | |
|---|---|
| Seed stocks | amiRCWC15<br>amiRCWC15/pPAG1::gPAG1-FM<br>pCWC15::GFP-CWC15/amiRCWC15 |
| Novel plant genotypes | amiRCWC15 plants were produced by Agrobacterium (harbouring pBTEX1300-amiRCWC15 vector)-mediated transformation in Col background. At least three independent lines were analyzed in the related experiments in Fig.1, 2, 4, 5, 6 and 7.<br>amiRCWC15/pPAG1::gPAG1-FM plants were produced by Agrobacterium (harbouring pBTEX1300-amiRCWC15 vector)-mediated transformation in pPAG1::gPAG1-FM plants. At least three independent lines were analyzed in the related experiment of Fig. 5.<br>pCWC15::GFP-CWC15/amiRCWC15 plants were produced by Agrobacterium (harbouring pCWC15::GFP-CWC15 vector)-mediated transformation in amiRCWC15 plants. At least two independent lines were analyzed in the related experiments of Fig. 2 and 3. |
| Authentication | amiRCWC15 and amiRCWC15/pPAG1::gPAG1-FM lines all displayed pleiotropic development defects such as smaller plant size and serrated leaves. In T1 generation, we identified at least three amiRCWC15 independent lines, in which the transcript levels of CWC15 were reduced, showing that they are indeed the knockdown lines of CWC15 gene. Expression of a CWC15 open reading frame fused with a GFP tag at its C-terminal driven by its native promoter (pCWC15::GFP-CWC15) in amiRCWC15-1 line produced pCWC15::GFP-CWC15/amiRCWC15 plants and fully rescued the developmental defects of amiRCWC15 (SI Appendix, Fig. S3B), demonstrating that the growth defects of amiRCWC15 are caused by reduced CWC15 transcript levels. |

# ChIP-seq

## Data deposition

☐ Confirm that both raw and final processed data have been deposited in a public database such as GEO.

☐ Confirm that you have deposited or provided access to graph files (e.g. BED files) for the called peaks.

| | |
|---|---|
| Data access links<br>*May remain private before publication.* | *For "Initial submission" or "Revised version" documents, provide reviewer access links. For your "Final submission" document, provide a link to the deposited data.* |
| Files in database submission | *Provide a list of all files available in the database submission.* |
| Genome browser session<br>(e.g. UCSC) | *Provide a link to an anonymized genome browser session for "Initial submission" and "Revised version" documents only, to enable peer review. Write "no longer applicable" for "Final submission" documents.* |

## Methodology

| | |
|---|---|
| Replicates | *Describe the experimental replicates, specifying number, type and replicate agreement.* |
| Sequencing depth | *Describe the sequencing depth for each experiment, providing the total number of reads, uniquely mapped reads, length of reads and whether they were paired- or single-end.* |
| Antibodies | *Describe the antibodies used for the ChIP-seq experiments; as applicable, provide supplier name, catalog number, clone name, and lot number.* |
| Peak calling parameters | *Specify the command line program and parameters used for read mapping and peak calling, including the ChIP, control and index files used.* |
| Data quality | *Describe the methods used to ensure data quality in full detail, including how many peaks are at FDR 5% and above 5-fold enrichment.* |
| Software | *Describe the software used to collect and analyze the ChIP-seq data. For custom code that has been deposited into a community repository, provide accession details.* |

# Flow Cytometry

## Plots

Confirm that:

☐ The axis labels state the marker and fluorochrome used (e.g. CD4-FITC).

☐ The axis scales are clearly visible. Include numbers along axes only for bottom left plot of group (a 'group' is an analysis of identical markers).

☐ All plots are contour plots with outliers or pseudocolor plots.

☐ A numerical value for number of cells or percentage (with statistics) is provided.

## Methodology

| | |
|---|---|
| Sample preparation | *Describe the sample preparation, detailing the biological source of the cells and any tissue processing steps used.* |
| Instrument | *Identify the instrument used for data collection, specifying make and model number.* |
| Software | *Describe the software used to collect and analyze the flow cytometry data. For custom code that has been deposited into a community repository, provide accession details.* |
| Cell population abundance | *Describe the abundance of the relevant cell populations within post-sort fractions, providing details on the purity of the samples and how it was determined.* |
| Gating strategy | *Describe the gating strategy used for all relevant experiments, specifying the preliminary FSC/SSC gates of the starting cell population, indicating where boundaries between "positive" and "negative" staining cell populations are defined.* |

☐ Tick this box to confirm that a figure exemplifying the gating strategy is provided in the Supplementary Information.

# Magnetic resonance imaging

## Experimental design

| | |
|---|---|
| Design type | *Indicate task or resting state; event-related or block design.* |
| Design specifications | *Specify the number of blocks, trials or experimental units per session and/or subject, and specify the length of each trial or block (if trials are blocked) and interval between trials.* |
| Behavioral performance measures | *State number and/or type of variables recorded (e.g. correct button press, response time) and what statistics were used to establish that the subjects were performing the task as expected (e.g. mean, range, and/or standard deviation across subjects).* |

## Acquisition

| | |
|---|---|
| Imaging type(s) | *Specify: functional, structural, diffusion, perfusion.* |
| Field strength | *Specify in Tesla* |
| Sequence & imaging parameters | *Specify the pulse sequence type (gradient echo, spin echo, etc.), imaging type (EPI, spiral, etc.), field of view, matrix size, slice thickness, orientation and TE/TR/flip angle.* |
| Area of acquisition | *State whether a whole brain scan was used OR define the area of acquisition, describing how the region was determined.* |

Diffusion MRI ☐ Used ☐ Not used

## Preprocessing

| | |
|---|---|
| Preprocessing software | *Provide detail on software version and revision number and on specific parameters (model/functions, brain extraction, segmentation, smoothing kernel size, etc.).* |
| Normalization | *If data were normalized/standardized, describe the approach(es): specify linear or non-linear and define image types used for transformation OR indicate that data were not normalized and explain rationale for lack of normalization.* |
| Normalization template | *Describe the template used for normalization/transformation, specifying subject space or group standardized space (e.g. original Talairach, MNI305, ICBM152) OR indicate that the data were not normalized.* |
| Noise and artifact removal | *Describe your procedure(s) for artifact and structured noise removal, specifying motion parameters, tissue signals and physiological signals (heart rate, respiration).* |
| Volume censoring | *Define your software and/or method and criteria for volume censoring, and state the extent of such censoring.* |

## Statistical modeling & inference

| | |
|---|---|
| Model type and settings | *Specify type (mass univariate, multivariate, RSA, predictive, etc.) and describe essential details of the model at the first and second levels (e.g. fixed, random or mixed effects; drift or auto-correlation).* |
| Effect(s) tested | *Define precise effect in terms of the task or stimulus conditions instead of psychological concepts and indicate whether ANOVA or factorial designs were used.* |

Specify type of analysis: ☐ Whole brain ☐ ROI-based ☐ Both

Statistic type for inference

(See Eklund et al. 2016)

*Specify voxel-wise or cluster-wise and report all relevant parameters for cluster-wise methods.*

Correction

*Describe the type of correction and how it is obtained for multiple comparisons (e.g. FWE, FDR, permutation or Monte Carlo).*

## Models & analysis

| n/a | Involved in the study |
| --- | --- |
| ☐ | ☐ Functional and/or effective connectivity |
| ☐ | ☐ Graph analysis |
| ☐ | ☐ Multivariate modeling or predictive analysis |

Functional and/or effective connectivity

*Report the measures of dependence used and the model details (e.g. Pearson correlation, partial correlation, mutual information).*

Graph analysis

*Report the dependent variable and connectivity measure, specifying weighted graph or binarized graph, subject- or group-level, and the global and/or node summaries used (e.g. clustering coefficient, efficiency, etc.).*

Multivariate modeling and predictive analysis

*Specify independent variables, features extraction and dimension reduction, model, training and evaluation metrics.*
